# Enhancing Sensitivity of Commercial Gold Nanoparticle-Based Lateral Flow Assays: A Comparative Study of Colorimetric and Photothermal Approaches

**DOI:** 10.3390/s25164904

**Published:** 2025-08-08

**Authors:** Jully Blackshare, Hansel A. Mina, Amanda J. Deering, Bartek Rajwa, J. Paul Robinson, Euiwon Bae

**Affiliations:** 1 Applied Optics Laboratory, School of Mechanical Engineering, Purdue University, West Lafayette, IN 47907, USA; lee4107@purdue.edu; 2 Department of Food Science, Purdue University, West Lafayette, IN 47907, USAadeering@purdue.edu (A.J.D.); 3 Bindley Bioscience Center, Discovery Park, Purdue University, West Lafayette, IN 47907, USA; brajwa@purdue.edu; 4 Department of Basic Medical Sciences, Purdue University, West Lafayette, IN 47907, USA; 5 Weldon School of Biomedical Engineering, Purdue University, West Lafayette, IN 47907, USA

**Keywords:** lateral flow assays, foodborne pathogens, *Salmonella* detection, colorimetric analysis, photothermal speckle imaging, smartphone-based biosensing, machine learning

## Abstract

Lateral flow assays (LFAs) are extensively utilized in point-of-care diagnostics due to their affordability, simplicity, and rapid time-to-results. However, their low sensitivity remains a significant limitation, particularly for detecting foodborne pathogens at concentrations below regulatory thresholds. This study evaluated two distinct sensing modalities—photothermal speckle imaging and colorimetric line intensity analysis—for their potential to enhance the sensitivity of commercially available LFAs. Photothermal imaging quantified refractive index shifts induced by plasmonic heating of gold nanoparticles, while colorimetric analysis used smartphone-acquired images processed with machine learning. The photothermal method achieved a limit of detection (LOD) of 2.13 × 10^5^ CFU/mL, while the colorimetric approach, using a logistic regression model with LASSO regularization, achieved an LOD of 10^5^ CFU/mL. While both approaches demonstrated detection thresholds comparable to traditional visual interpretation, the colorimetric method provided an added advantage by enabling quantitative prediction of bacterial concentration through regression modeling. With further optimization of each sensing method, these findings demonstrate the feasibility of improving unmodified commercial LFAs through optical and computational enhancements, offering a promising pathway toward the development of portable biosensing systems for real-time food safety monitoring.

## 1. Introduction

Food safety is a critical determinant of public health and quality of life. The consumption of food contaminated with pathogenic microorganisms can cause serious illness, posing a global health concern across both developed and developing nations. Foodborne diseases affect approximately 600 million people annually, with one in four individuals in the United States experiencing such illnesses each year [1,2]. Beyond health impacts, foodborne pathogens impose a significant economic burden on healthcare systems [3,4,5]. While gold-standard methods for pathogen detection, such as culture-based techniques and polymerase chain reactions (PCR), provide high accuracy, they are time-consuming and labor-intensive [6]. Symptom onset depends on the specific toxic ingested, underscoring the importance of early detection prior to product distribution [3,7]. However, the prolonged turnaround time of conventional methods limits their applicability in rapid food safety monitoring.

To address these limitations, rapid diagnostic tools such as lateral flow assays (LFAs) have been developed. While widely utilized, LFAs suffer from a critical limitation, which is their low sensitivity. High bacterial concentrations are typically required to yield positive results, while samples with lower concentration levels may lead to false-negatives or require enrichment steps [8,9,10,11,12]. This sensitivity gap poses a challenge for applications requiring detection at or below regulatory thresholds set by agencies like the United States Department of Agriculture (USDA) [13].

Spherical gold nanoparticles (AuNPs) are commonly used tracers in standard LFAs. Colorimetric analysis, widely utilized to enhance LFA sensitivity, amplifies the test line intensity through imaging. Further advancements have been achieved by incorporating image processing and custom algorithms that enhance the contrast between the test line and membrane background. to optimize test line detection [14,15,16,17,18]. Optical components such as a charge-coupled device (CCD) camera have been used to detect creatine kinase. The Bayer filters were used to increase selectivity by targeting specific wavelengths defined by the test line’s color [15,16,17]. Similarly, complementary metal-oxide semiconductor (CMOS)-based portable reader was developed to analyze a commercial LFA designed for neutrophil elastase (NE) detection [18].

Advancements in smartphone technology have enabled mobile-based biosensing platforms [19]. These systems utilize camera-based imaging and image processing to analyze test line intensity. Calibration curves can be generated, enabling quantitative assessment as well [20,21]. Various color spaces have been explored to improve test line detection [22]. For food pathogen detection, colorimetric analysis lowered the limit of detection (LOD) for *Escherichia coli* O157 to 500 CFU/mL [23]. However, such improvements often rely on modified tracers such as gold-decorated polystyrene particles, which exhibit enhanced optical properties.

Despite these advancements, conventional AuNPs still struggle to generate sufficient signal for food pathogen monitoring. Research has therefore focused on modifying tracers or assay structures to increase signal intensity [24,25,26]. However, these modifications can be costly and may lack the stability of commercially produced AuNPs [27,28]. An alternative approach involves the use of machine learning. For example, machine learning algorithms trained on LFA images have lowered the LOD of COVID-19 assays by one order of magnitude [29,30].

Another promising technique involves measuring the thermal response of the membranes. Surface plasmon resonance (SPR), widely studied since the 1990s, has enabled tunable optical properties through engineered metasurfaces made of graphene or Dirac semimetals. These structures provide high absorption and angle-insensitive performance [31,32,33,34]. In pathogen detection, the SPR properties of AuNPs can be exploited for thermal contrast imaging. When irradiated with light at their resonance plasmon wavelength, particles generate heat [35]. For instance, a 532 nm laser induced localized heating in nanoparticles, leading to temperature changes which were measured using thermal cameras in prior studies [36,37]. FDA-approved LFAs for cryptococcal antigen detection using thermal contrast methods achieved a LOD 32 times lower than traditional colorimetric analysis [38]. A photothermal card reader was also developed to quantify 13 different food hazards, demonstrating the potential of this technique in food safety applications [39].

Heat signals can be quantified using various approaches. For example, a microfiber long-period grating (mLPG) measured photothermal activity using thermo-photonic lock-in imaging [40,41]. Similarly, photothermal imaging quantifies heat signal by tracking refractive index changes caused by localized heating. These changes affect light propagation and appear as variations in speckle patterns or intensity fluctuations [42].

Photothermal sensing has demonstrated remarkable improvements in LFA sensitivity for food pathogen detection. However, like colorimetric methods, it often relies on modified tracers to optimize plasmonic properties. Although silver nanoparticles (AgNPs) generate more heat than gold, AuNPs remain preferred due to their superior stability [43]. Ultimately, further research is needed to improve sensitivity without requiring custom laboratory-made assays.

In this study, we compare two distinct sensing modalities—colorimetric analysis with machine learning and photothermal speckle imaging—for their effectiveness in detecting *Salmonella*. While colorimetric analysis leverages image processing and machine learning to improve test line detection and quantification, photothermal speckle imaging detects refractive index changes through speckle pattern shifts. By evaluating these two methodologies, this study provides valuable insights into their strengths, limitations, and practical applicability, contributing to the advancement of LFA-based pathogen detection. Although individual sensing techniques are well established, we aim to directly compare and integrate these approaches to build a foundation for a unified, portable detection system. Specifically, we assess whether either method can achieve lower or more reliable LODs for *Salmonella* detection without modifying the assay architecture.

## 2. Materials and Methods

### 2.1. Sample Preparation

#### 2.1.1. AuNP Membrane

Gold nanoparticles with a diameter of 80 nm (G-80-20, Cytodiagnostics, Burlington, ON, Canada) were used for calibration. The stock solution with an initial concentration of 7.82 × 10^9^ NPs/mL was concentrated by centrifuging 100 mL at 600× *g* for 30 min. The supernatant was discarded and 780 µL of phosphate-buffered saline (PBS) was added, yielding a final concentration of approximately 10^12^ NPs/mL. The concentrated solution was serially diluted, and five microliters from each dilution were dispensed onto a nitrocellulose membrane. Membranes were subsequently dried in a chemical hood for 10 min.

#### 2.1.2. Salmonella-Spiked Sample

**Figure 1:**
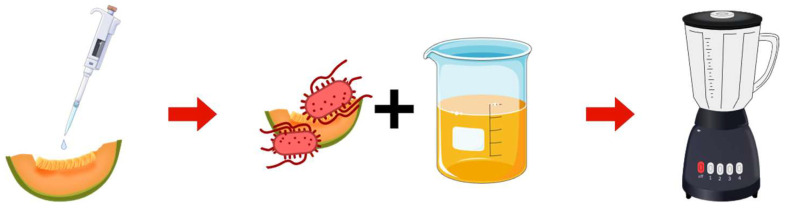
BAM-based sample preparation workflow. *Salmonella* culture is spot-inoculated on cantaloupe and incubated at 4 °C overnight. The cantaloupe is mixed with sterile PBS. The mixture is blended and transferred to a sterile jar for further incubation.

A commercially available lateral flow assay for *Salmonella* detection (STLF-020, BioAssay Works, Ijamsville, MD, United States) was used with cantaloupe as the food matrix. The testing procedure followed the Bacteriological Analytical Manual (BAM) guidelines established by the U.S. Food and Drug Administration (FDA) [44]. The sample preparation workflow is illustrated in Figure 1.

First, 25 g of cantaloupe were spot inoculated with 1 mL of *Salmonella enterica* ser. Typhimurium ATCC 14028 culture to obtain concentrations of 10^4^ to 10^7^ CFU/mL. After drying in a biosafety cabinet for 2 h, samples were stored overnight at 4 °C. The inoculated cantaloupe was mixed with 225 mL of phosphate-buffer solution (PBS). The mixture was transferred into a sterile blender jar (NINJA^®^, BL660, Needham, MA, United States) and homogenized for 1 min. The resulting homogenates were serially diluted in PBS, and viable counts were determined by plating on XLT-4 plates according to the guidelines in the Bacteriological Analytical Manual (BAM). Samples inoculated with *Salmonella* used for the assay were heat-inactivated at 100 °C for 10 min to ensure safety, then cooled to room temperature. All assays were performed per the manufacturer’s instructions.

### 2.2. Photothermal Speckle Imaging

#### 2.2.1. System Design

A benchtop system was designed to observe the photothermal activity of AuNPs. Given that the plasmon resonance wavelength of AuNPs is approximately 520 nm, a 532 nm laser (PGL-532, Edmund Optics, Barrington, NJ, United States) was selected to induce localized heating [24]. A 30 mW 780 nm laser (401, CivilLaser, Hangzhou, China) illuminated the nitrocellulose membrane. As coherent light interacts with an inhomogeneous surface, speckle patterns are formed on the imaging screen. This specific wavelength was chosen based on its demonstrated effectiveness in prior studies using similar setups for analogous applications [42].

**Figure 2:**
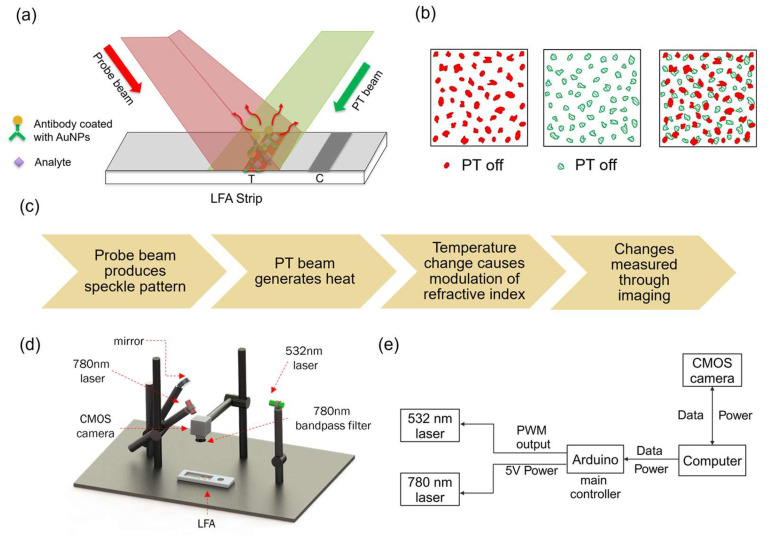
Schematic of the photothermal speckle imaging system. (**a**) Illustration of laser irradiation on the LFA test strip. The probe beam (780 nm) generates a speckle pattern, while the photothermal (PT) beam (532 nm) excites the nanoparticles. (**b**) The localized heating induced by nanoparticle excitation alters the refractive index of the membrane, leading to a measurable shift in speckle patterns. (**c**) Workflow of the photothermal speckle imaging system, detailing the sequential processes involved in speckle pattern acquisition and analysis. (**d**) Schematic diagram of the benchtop system, with labeled components. (**e**) Control diagram of the benchtop system, illustrating the communication between system components.

As illustrated in Figure 2a, antibody-conjugated AuNPs were excited by the photothermal (532 nm) laser, while the continuous probe (780 nm) beam produced the speckle pattern. The modulation rate set to 1 Hz was controlled via Arduino. The induced temperature change altered the membrane’s local refractive index, resulting in detectable speckle shifts (Figure 2b). To isolate probe light, a 780 nm bandpass filter (FBH780-10, ThorLabs, Newton, NJ, United States) was placed in front of the CMOS camera (Aca4600-10uc, Basler, Exton, PA, United States). Images were recorded at 30 frames per second in bitmap format, capturing the laser-illuminated region. A total of 300 frames were collected per experiment. The overall workflow of the system is outlined in Figure 2c, while Figure 2d,e show the schematic of the system and the control diagram illustrating the communication between system components.

A 532 nm laser was used to continuously illuminate the AuNP-deposited membrane, and the temperature change was recorded using an infrared (IR) camera (TI300+ 60 HZ, Fluke Corporation, Everett, WA, United States). Negative control, consisting of a membrane with PBS, was used as a baseline measurement. The temperature changes were calculated by subtracting control readings from those of AuNP-spotted membranes.

#### 2.2.2. Signal Processing

Each pixel’s intensity was extracted from the acquired images over time. After DC gain removal, a Fast Fourier Transform (FFT) was applied to convert temporal signals to the frequency domain. The signal magnitude was then averaged across all frames. The final processed data was plotted, revealing a peak magnitude at the modulation frequency. This value represented the relative AuNP activity at the test line.

### 2.3. Colorimetric Analysis

#### 2.3.1. Line Intensity Calculation

**Figure 3:**
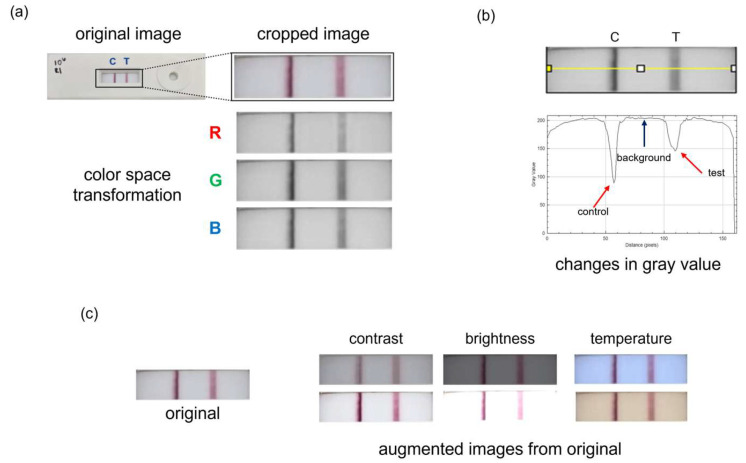
Colorimetric analysis and data augmentation. (**a**) The LFA image is cropped to isolate the region of interest, encompassing the test and control lines. It is then split into the RGB color space for further analysis. (**b**) The yellow line indicates the region where grayscale intensity values are extracted. The corresponding plot below shows intensity variations, with dips representing the control and test lines. (**c**) Visual representation of the data augmentation process. Three image parameters—temperature, contrast, and brightness—are varied from negative to positive values to simulate diverse imaging conditions and expand the dataset for machine learning training.

Test line intensity was analyzed from LFA images captured using a Samsung S24 smartphone (Samsung Electronics America, Ridgefield Park, NJ, USA). The camera was positioned perpendicular to the strip, and imaging was performed under uniform white LED within a light box to minimize lighting variability. All images were acquired using default camera settings and no manual adjustments were made to exposure or ISO. Auto-focus and auto-white balance features were enabled throughout image acquisition. As illustrated in Figure 3a, the images were transformed into RGB color space. Analysis was performed in ImageJ (version 1.54j, National Institutes of Health, Bethesda, MD, USA) using a color transformation plugin and the “Plot Profile” tool to extract grayscale values along the defined line (Figure 3b) [45,46]. Higher values correspond to lighter colors, while lower values indicate darker regions. To account for variations in lighting conditions across images, intensity values were selected by identifying the minimum grayscale value within the test line region and the maximum grayscale value of the background. The test line intensity,

(1) $$I_{test,norm}=\frac{I_{test}}{I_{background}}$$

was then normalized by dividing the test line intensity ($I_{test}$) by the background intensity ($I_{background}$). This process was repeated using a negative control sample ($I_{PBS,norm}$), where the absence of a test line results in a normalized intensity close to 1. Lastly, the ratio of the test line intensity between the positive and negative samples,

(2) $$I_{test/PBS}=\frac{I_{test,norm}}{I_{PBS,\;norm}}$$

was calculated. A sample is classified as positive if the computed ratio exceeds 1.

#### 2.3.2. Data Augmentation

To expand the training dataset, data augmentation techniques were employed. As seen in Figure 3c, three image parameters were varied: brightness, contrast, and temperature. Brightness and contrast values were adjusted from −25 to +25 in increments of 1, while temperature was modified from −10 to +10 in increments of 1, resulting in a dataset comprising 16,000 images for machine learning analysis. In addition to increasing the dataset size, these augmentations simulated realistic variability, enhancing model robustness.

The dataset was split into 80% for training and 20% for testing. Three different regression models were employed to predict bacterial concentration based on colorimetric intensity values: linear, polynomial (degree = 2), and logistic regression. Least Absolute Shrinkage and Selection Operator (LASSO) regularization was applied to each model to assess its effect on predictive performance and model complexity. Target labels were approximated from optical measurements and reflect order of magnitude groupings.

## 3. Results

### 3.1. AuNPs Calibration

To investigate the correlation between AuNPs (d = 80 nm) concentration and photothermal response, nitrocellulose membranes were spotted with AuNPs at three concentrations. While our study employed a serially diluted set of three concentrations to demonstrate the trend, a more comprehensive concentration-effect analysis has been reported in previous work. It examined AuNP concentrations ranging from 10^9^ to 10^12^ NPs/mL and demonstrated a strong correlation between nanoparticle concentration and thermal response [36]. In our study, the selected concentrations served as calibration reference to validate the principle that increasing AuNP concentration results in greater photothermal response.

**Figure 4:**
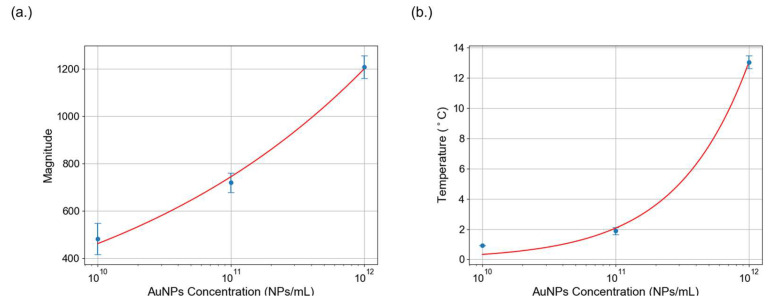
Calibration of AuNPs using temperature and photothermal signal analysis. (**a**) The relationship between temperature change and AuNP concentration is plotted. The fitted exponential model illustrates the correlation between nanoparticle concentration and nanoparticle heating. (**b**) Calibration curve of the FFT-processed photothermal signal, where the peak magnitude is plotted against AuNP concentrations. The signal magnitude decreases with lower AuNP concentrations, like the temperature data.

Figure 4a presents the temperature variations at different concentrations measured using the Ti300+ infrared camera. Each data point represents the average of three replicates. The results revealed a positive correlation as higher nanoparticle concentrations produced greater thermal response. The plotted calibration curve further illustrates this relationship, revealing a significant drop in temperature change as the concentration of AuNPs is reduced.

In addition to temperature measurements, speckle pattern analysis was performed under 532 nm laser excitation modulated at 2 Hz, with a continuous 780 nm probe beam. FFT analysis of speckle intensity yielded a spectral peak at the modulation frequency, confirming the photothermal activity. Three independent datasets were acquired for each concentration, and the calibration curve is represented. In Figure 4b, signal magnitude decreased with lower AuNP concentrations, consistent with the temperature trends. These results validate the photothermal effect of AuNPs and their suitability for photothermal speckle analysis.

### 3.2. Salmonella Detection

#### 3.2.1. Photothermal Speckle Analysis

**Figure 5:**
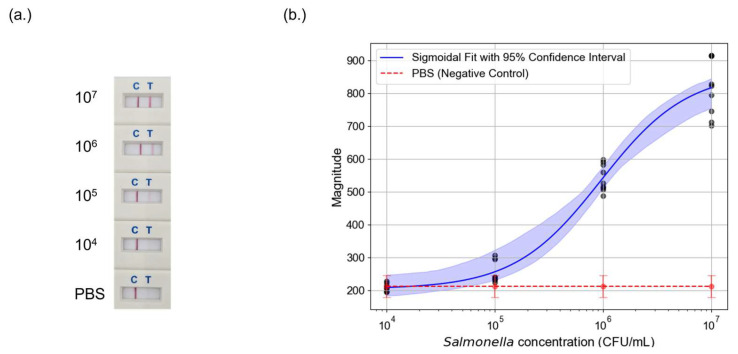
Detection of *Salmonella* using LFAs and photothermal speckle imaging. (**a**) LFA test strips at varying *Salmonella* counts. The test line becomes fainter as the bacterial concentration decreases, making visual detection increasingly difficult. (**b**) Calibration curve of *Salmonella* detection using photothermal speckle imaging. The sigmoidal fit (blue line) with 95% confidence interval (shaded blue region) illustrates the relationship between *Salmonella* concentration and photothermal signal magnitude. The PBS negative control (red dashed line) is plotted as a baseline.

After confirming the photothermal effect, *Salmonella*-spiked LFAs were tested to assess detection performance. Four concentrations were evaluated in triplicate across three independent tests. Figure 5a shows the colorimetric results of the LFA tests. The test line begins to fade at 10^5^ CFU/mL. At 10^4^ CFU/mL, both negative control and the test line were indistinguishable to the naked eye. This result highlights a key limitation of conventional AuNP-based LFAs, since they struggle to detect lower bacterial concentrations due to insufficient signal intensity.

Speckle analysis followed the same protocol as the calibration study. Figure 5b presents the corresponding speckle signal calibration curve fitted with a four-parameter sigmoidal model:

(3) $$y=\frac{\beta_{1}}{1+\exp\left(-\beta_{2}\left(x-\beta_{3}\right)\right)}+\beta_{4}$$

where

- $\beta_{1}$ controls the amplitude of the response;
- $\beta_{2}$ controls the steepness of the curve;
- $\beta_{3}$ represents the inflection points;
- $\beta_{4}$ is the baseline offset.

Signal intensity declined with decreasing *Salmonella* concentration. The red line in Figure 5b represents negative control, establishing the baseline. The 95% confidence interval is overlaid on the sigmoidal fit of *Salmonella* data, illustrating the variability in measurements across different trials. Notably, the system’s detection performance diminishes around 10^4^ CFU/mL, where the test line signal approaches the noise threshold. While photothermal speckle imaging provides improved sensitivity compared to colorimetric analysis, further optimization such as using advanced speckle analysis algorithms or higher-powered laser may be necessary to achieve robust detection at lower bacterial concentrations.

#### 3.2.2. RGB Color-Space Intensity Analysis

**Figure 6:**
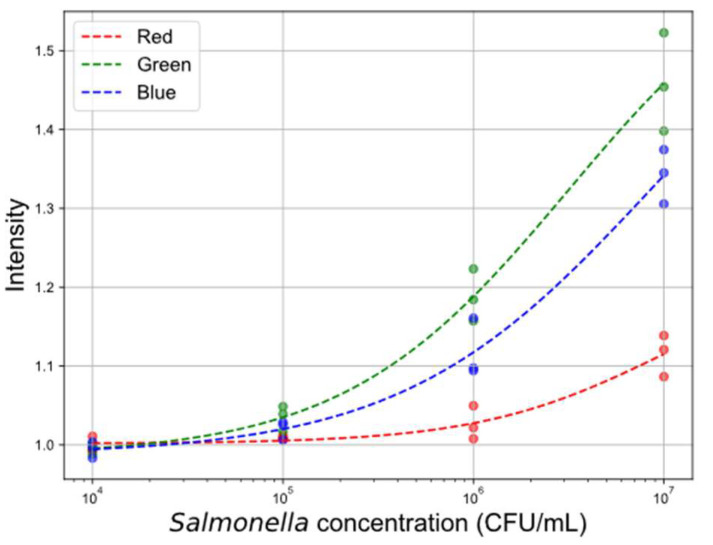
Calibration curves for line intensity data across different color channels. The plot illustrates the relationship between *Salmonella* concentration and normalized line intensity values for the red, green, and blue color channels.

To quantify colorimetric response, line intensities were extracted from RGB channels. Each concentration yielded three replicate values per channel. A four-parameter logistic (4PL) regression model, a standard nonlinear univariate calibration method in immunoassay analysis, was used [47]:

(4) $$f\left(x\right)=\beta_{2}+\frac{\beta_{1}-\beta_{2}}{1+{\left(\frac{x}{\beta_{3}}\right)}^{-\beta_{4}}\;}$$

where *x* represents concentration. $\beta_{1}$ and $\beta_{2}$ are the lower and upper asymptotes. $\beta_{3}$ is the inflection point and $\beta_{4}$ is related to the slope. This analysis served to assess the individual predictive contributions of each color channel prior to the application of machine learning models. The results are shown in Figure 6.

All channels exhibited declining intensity with decreasing concentration. The green channel demonstrated the strongest response, attributed to the Bayer filter design in most camera sensors, which includes twice as many green pixels compared to red and blue.

#### 3.2.3. Machine Learning Regression Analysis

Three regression models were developed to evaluate their performance in predicting bacterial concentration: linear, polynomial, and multivariate logistic regression. All models were implemented using the SciPy library in Python (version 3.13.2, Python Software Foundation, Wilmington, DE, USA) [48]. For linear regression, the following model was constructed:

(5) $$y=\beta_{0}+\beta_{1}\cdot \log\left(Red\right)+\beta_{2\cdot }\log\left(Green\right)+\beta_{3}\cdot log(Blue)$$

where the log-transformed intensity values from the red, green, and blue colors channels were used as predictors. Here, y represents the predicted bacterial concentration, $\beta_{0}$ is the intercept, and $\beta_{1},\;\beta_{2},\beta_{3}$ are the regression coefficients.

For polynomial regression, a second-degree multivariate model was built:

(6) $$\begin{array}{c} y=\beta_{0}+\beta_{1}\log\left(Red\right)+\beta_{2}\log\left(Green\right)+\beta_{3}\log\left(Blue\right)+\beta_{4}{\log\left(Red\right)}^{2}\\ +\beta_{5}\log\left(Red\right)\log\left(Green\right)+\beta_{6}\log\left(Red\right)\log\left(Blue\right)\\ +\beta_{7}\log{\left(Green\right)}^{2}+\;\beta_{7}{\log\left(Green\right)}^{2}\\ +\beta_{8}\log\left(Green\right)\log\left(Blue\right)+\beta_{9}\log{\left(Blue\right)}^{2} \end{array}$$

As with the linear model, $\beta_{0}$ is the intercept, and $\beta_{1}$ through $\beta_{9}$ are the corresponding regression coefficients.

Finally, a multivariate logistic regression model was applied:

(7) $$y=\frac{\beta_{1}}{1+\exp\left(-\left(\beta_{2}x_{1}+\beta_{3}x_{2}+\beta_{4}x_{3}+\beta_{5}\right)\right)}$$

where y is the predicted concentration, and $x_{1},\;x_{2},x_{3}$ corresponds to the log-transformed red, green, and blue channel intensities, respectively. The parameters $\beta_{1}$ through $\beta_{5}$ are the model coefficients. This form of the logistic model was selected for its multivariate structure to capture nonlinear relationships across multiple input channels, in contrast to traditional univariate sigmoidal models.

##### Baseline Model Performance Without Regularization

**Figure 7:**
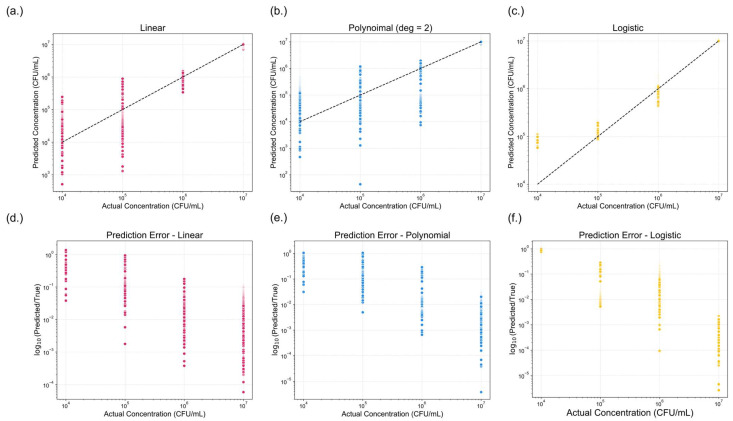
Comparison of model performance across three regression approaches. (**a**–**c**) presents the predicted versus actual concentrations (CFU/mL) for (**a**) linear, (**b**) polynomial (degree = 2), and (**c**) logistic regression models. The dotted black line represents the ideal prediction. (**d**–**f**) shows the corresponding prediction errors, expressed as the logarithmic fold change (log_10_(predicted/true)) for (**d**) linear, (**e**) polynomial (degree = 2), and (**f**) logistic models. A lower log-fold change indicates reduced prediction error. Among the three models, logistic regression demonstrated the best predictive performance, exhibiting the smallest overall error.

Logistic regression model exhibited superior performance compared to the linear and polynomial models as shown in Figure 7. All models performed well at higher concentrations but showed declining accuracy below 10^6^ CFU/mL. Models showed substantial errors at 10^5^ CFU/mL and lower. The logistic model outperformed the others, maintaining narrower prediction ranges at 10^4^–10^5^ CFU/mL.

Log-fold errors were calculated rather than traditional residuals or percent errors, as they primary goal was to predict the bacterial concentration on a 10× scale. In food safety applications, the order of magnitude of bacterial concentration is of greater significance than minor variations in numerical value. Thus, log-fold change provides a more meaningful assessment of predictive accuracy. From the error analysis, it was observed that for samples with an actual concentration of 10^4^ CFU/mL, the logistic model consistently predicted values between 10^4^ and 10^5^ CFU/mL, whereas the linear and polynomial models often produced predictions spanning from 10^4^ to 10^6^ CFU/mL. This narrower prediction range further highlights the superior performance of the logistic model.

Table 1 summarizes the quantitative evaluation of each regression model. Given that conventional coefficient of determination (R^2^) is not applicable for logistic regression, McFadden’s pseudo-R^2^ was chosen for its simplicity. It is defined as:

(8) RMcF2=1−lnLmodellnLnully

where $\ln L_{model}$ is the log-likelihood of the fitted model, and $\ln L_{null}$ is the log-likelihood of the null model. This metric evaluates the relative improvement of the fitted model over the null model, quantifying how well the model explains the observed data [49]. Pseudo-R^2^ values tend to yield lower values than traditional R^2^, though ranging between 0.2 and 0.4 for models are considered to have acceptable predictive performance [50]. Among three models, only the logistic regression achieved a pseudo-R^2^ value exceeding 0.2. Bootstrapping with 1000 iterations yielded 95% confidence intervals for the pseudo-R^2^ values: [0.1272, 0.1287] for the linear model, [0.1448, 0.1461] for the polynomial model, and [0.2180, 0.2201] for the logistic model. Additionally, model complexity was assessed using the Akaike Information Criterion (AIC) and the Bayesian Information Criterion (BIC), both of which penalize models for unnecessary complexity [51,52]. Lower AIC and BIC values indicate better model simplicity. Across all three metrics—pseudo-R^2^, AIC, and BIC—the logistic regression model consistently outperformed the linear and polynomial models. Thus, logistic regression was identified as the most suitable model for predicting bacterial concentration in this study.

To address potential overfitting concerns, we implemented five-fold cross-validation for each model. Evaluation metrics were averaged across folds to assess model stability. For non-regularized models, pseudo-R^2^ was. 0.1280 for linear regression, 0.1455 for polynomial regression, and 0.2190 for the sigmoidal model. All models demonstrated narrow confidence intervals and low standard deviations, indicating consistent and reliable performance across folds.

##### Model Performance After LASSO Regularization

LASSO regularization was applied to assess its impact on model performance and variable selection. LASSO introduces an 𝓁_1_ penalty term that encourages sparsity in the model by shrinking less significant regression coefficients toward zero, thereby reducing model complexity and potentially improving generalization [53]. The LASSO optimization model for linear regression can be written as:

(9) $${\hat{\beta }}^{LASSO}=arg\underset{\beta_{0},\beta }{min}\;\left\{\frac{1}{2n}\sum_{i=1}^{n}\;{\left(y_{i}-\beta_{0}-f\left(x_{i};\beta \right)\right)}^{2}+\lambda \|\beta {\|}_{1}\right\}$$

In Equation (9),

- n is the number of training samples;
- $y_{i}$ is the observed target for the i-th sample;
- β is the vector of regression coefficients;
- $\beta_{0}$ is the intercept;
- λ is the regularization parameter that controls the strength of 𝓁_1_ penalty;
- $f\left(x_{i};\beta \right)$ encompasses:
  ◦ Linear regression: $f\left(x_{i};\beta \right)=x_{i}^{T}\beta$
  ◦ Polynomial regression: $f\left(x_{i};\beta \right)=\beta_{1}+\beta_{2}x_{i}+\beta_{3}x_{i}^{2}+\ldots$
  ◦ Logistic regression: $f\left(x_{i};\beta \right)=\frac{\beta_{1}}{1+\exp\left(-\left(\beta_{2}x_{1}+\beta_{3}x_{2}+\beta_{4}x_{3}+\beta_{5}\right)\right)}$

To implement LASSO, we used LassoCV from the sklearn.linear_model package, which performs LASSO regression while automatically selecting the optimal value of the regularization parameter λ through cross-validation [54].

**Figure 8:**
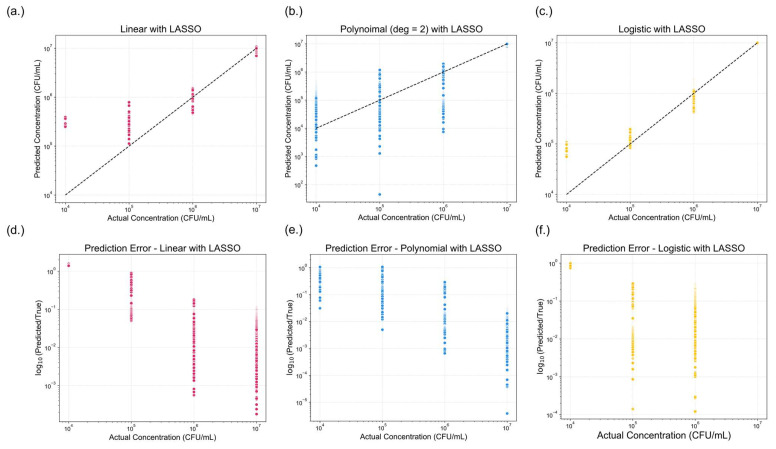
Comparison of model performance across three regression approaches with Least Absolute Shrinkage and Selection Operator (LASSO) regularization applied. (**a**–**c**) show the predicted versus actual concentrations (CFU/mL) for (**a**) linear with LASSO, (**b**) polynomial (degree = 2) with LASSO, and (**c**) logistic regression with LASSO. The dotted black line represents the ideal one-to-one correlation. (**d**–**f**) displays the corresponding prediction errors, expressed as the logarithmic fold change, for (**d**) linear, (**e**) polynomial (degree = 2), and (**f**) logistic regression models with LASSO. A lower-log fold change value indicates reduced prediction error.

Applying LASSO allowed for an evaluation of the relative importance of the color features and their contribution to model performance. Figure 8 presents the predictive performance of the three regression models after LASSO regularization. Compared to the base models, minimal changes in predictive accuracy were observed, as reflected in the performance metrics summarized in Table 2. The pseudo-R^2^ values remained mostly unchanged, with 95% confidence intervals for the linear, polynomial, and logistic models measured as [0.1253, 0.1267], [0.1358, 0.1371], and [0.2164, 0.2185], respectively. However, both AIC and BIC values decreased across all models, which is consistent with the expected outcome of model simplification following LASSO application. Five-fold cross-validation was also applied to each LASSO-regularized regression model to evaluate performance stability. The pseudo-R^2^ values remained consistent with those reported in Table 2, and all evaluation metrics exhibited low standard deviations and narrow confidence intervals, suggesting stable and reliable model behavior. Although LASSO did not markedly enhance predictive performance, it reduced model complexity. In all cases, the logistic model continued to outperform the linear and polynomial models, maintaining superior predictive performance.

#### 3.2.4. Comparison of Detection Limits

**Figure 9:**
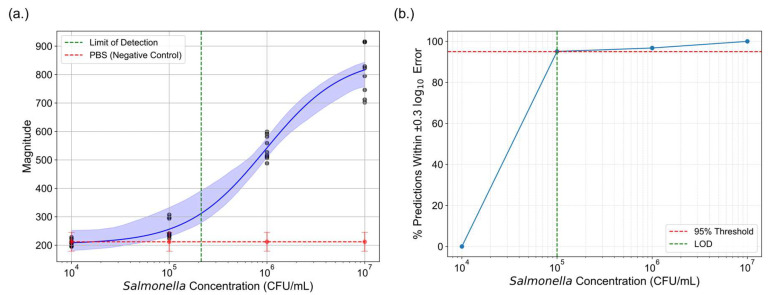
Comparison of the LOD between photothermal speckle imaging and machine learning assisted colorimetric analysis. (**a**) The green dashed line indicates the LOD for photothermal speckle imaging. (**b**) For colorimetric analysis, LOD was determined using a logistic regression model with LASSO regularization. The percentage of predictions falling within ${\pm 0.3log}_{10}$-fold error was calculated across concentrations. The LOD was defined as the lowest concentration at which ≥95% of predictions fall within this error margin, denoted by the intersection of the blue curve and red threshold line.

By exploring two distinct approaches to enhance the sensitivity of LFAs, we determined the limit of detection (LOD) for both photothermal speckle imaging and colorimetric analysis. For photothermal speckle imaging, the LOD was established by calculating the mean magnitude of the negative control and adding three times the standard deviation, a common statistical approach for determining detection thresholds. Based on this methodology, the LOD for speckle analysis was determined to be 2.12 × 10^5^ CFU/mL (Figure 9a).

To determine the LOD for colorimetric analysis, a logistic regression model with LASSO regularization was used. The ${log}_{10}$-fold change between predicted and actual concentrations was calculated for each test point. A prediction was considered acceptable if the error fell within a ±0.3 ${log}_{10}$ range, corresponding to a 2-fold difference. This threshold is commonly used in diagnostic studies as a biologically meaningful margin of error for concentration estimation [55,56]. As shown in Figure 9b, the percentage of predictions falling within this threshold was calculated for each tested concentration. The LOD was defined as the lowest concentration at which at least 95% of predictions satisfied this ${\pm 0.3log}_{10}$-fold error criterion. Based on this definition, the LOD for the machine learning-based colorimetric analysis was determined to be 10^5^ CFU/mL.

## 4. Discussion

This study compared two different sensing modalities—colorimetric analysis with machine learning and photothermal speckle imaging—for their ability to improve the sensitivity of LFAs in detecting *Salmonella*. While both methods achieved LODs comparable to visual interpretation, the colorimetric approach with machine learning demonstrated slightly improved quantitative prediction. These results provide a comparative assessment of each technique’s strengths and limitations, offering practical insight into their potential use in food safety diagnostics. To better contextualize the optical performance observed in both sensing methods, it is important to consider the morphological and optical characteristics of AuNPs used in the assays.

### 4.1. Morphological and Optical Characterization of AuNPs

The localized surface plasmon resonance (LSPR) of metallic nanoparticles is strongly influenced by their morphological characteristics, including shape, size and dielectric properties. Even among nanoparticles made of sample material, variations in size or geometry can shift the resonance wavelength [57,58]. For AuNPs, the peak absorption typically falls between 520 nm and 600 nm, depending on the particle diameter. As the size decreases, the solution color becomes lighter and the absorption peak shifts toward shorter wavelengths. In contrast, non-spherical shapes such as stars or popcorn-like structure exhibit distinct optical properties and appear in colors different from the characteristic red of spherical particles [24].

Characterizing the tracer particles is essential for accurate interpretation because LSPR behavior directly affects photothermal and colorimetric response. Calibration experiments were conducted using spherical AuNPs with a known diameter of 80 nm. The LFAs used for *Salmonella* detection did not disclose the physical properties of the gold-conjugated antibody tracers. Based on the vivid red color of the test line and the widespread use of spherical AuNPs in LFA manufacturing due to their optical stability, it was assumed that the embedded nanoparticles were standard spheres. While the exact size remains unknown, a 532 nm laser was selected as the most suitable option among commonly available laser wavelengths, as it aligns well with the typical absorption range of spherical AuNPs.

### 4.2. Sensitivity and Performance

The LOD for photothermal speckle imaging was determined to be 2.13 × 10^5^ CFU/mL, while LOD for the machine learning-assisted colorimetric analysis was approximately 10^5^ CFU/mL. Both methods converged to a similar detection threshold, aligning closely with the visual detection limits typically observed for AuNP-based LFAs targeting *Salmonella.* While the colorimetric method demonstrated a slightly lower LOD, the improvement over traditional visual interpretation was modest. Importantly, the LODs reported here approach the upper bound of detection limits observed in visually interpretable LFAs incorporating non-traditional AuNP enhancements. Reported visual LODs for these non-traditional AuNPs-based LFAs in *Salmonella* detection typically range from 10^3^ to 10^5^ CFU/mL. Such modifications may include surface functionalization or nanoparticle shape optimization to improve signal intensity and sensitivity [59,60,61].

It should be noted that the regression models were trained using discrete concentration levels ranging from 10^4^ to 10^7^ CFU/mL, treated as categorical targets. This approach, while practical, limited the model’s exposure to intermediate concentration values. Nevertheless, the use of regression rather than classification allowed the system to interpolate and predict concentrations between the tested values. Despite this limitation, the logistic regression model demonstrated robust and stable performance across the evaluated metrics. The pseudo-R^2^ values remained within acceptable ranges, and prediction errors were consistently low when compared to linear and polynomial models.

Although LASSO regularization did not lead to a significant improvement in predictive performance, it contributed to a reduction in model complexity, as indicated by the lower AIC and BIC values. By penalizing less informative features, LASSO helped mitigate the risk of overfitting, particularly in more parameter-heavy models like polynomial and logistic regression. Given these findings, logistic regression-particularly when combined with LASSO regularization-appears to be a well-suited approach for modeling concentration-dependent responses in biological systems such as LFAs.

In contrast, photothermal speckle imaging demonstrated a comparable detection capability to traditional visual inspection but did not outperform the colorimetric method. While this method effectively captured refractive index modulations caused by AuNPs, its sensitivity was constrained by factors such as laser power, system noise, and the relatively subtle thermal effects generated at low concentrations. The generated heat from the AuNPs may have been sufficient at lower concentrations to induce a detectable refractive index shift.

In prior studies on photothermal-based LFA systems for *Salmonella*, reported LODs range from 10^2^ to 10^4^ CFU/mL. However, these results often relied on custom-fabricated assays that incorporated non-traditional nanoparticles with enhanced photothermal properties [62,63,64,65]. For example, a study employed metallic alternatives that exhibit stronger light-to-heat conversion efficiencies than spherical AuNPs. These materials require different excitation wavelengths, sometimes in the near-infrared range, to match their absorption peaks. While such systems demonstrate high sensitivity, they also present challenges related to synthesis complexity, reproducibility, and compatibility with existing commercial platforms.

To date, only a limited number of studies have explored methods for enhancing the sensitivity of commercially available or traditional spherical-AuNP-based LFAs. Even fewer have focused specifically on improving the performance of these assays for foodborne pathogen detection [36,38,66]. Among the limited literature, photothermal speckle imaging has largely been applied to LFAs targeting non-foodborne pathogens [42]. As a result, the application of speckle imaging for food safety diagnostics and monitoring remains largely unexplored. This limited body of work underscores both the novelty and the practical challenges associated with integrating speckle-based thermal signal analysis into a field-deployable diagnostic tool for foodborne pathogens.

### 4.3. Implications for Food Safety and Future Works

The findings of the study provide practical insights into potential strategies for improving foodborne pathogen detection using commercially available LFAs. While the colorimetric analysis did not dramatically lower the limit of detection compared to visual interpretation, its ability to provide quantitative outputs through machine learning models presents a compelling advantage. With further refinement, this approach could be adapted into portable, smartphone-based biosensing platforms capable for supporting rapid, on-site food safety monitoring [67,68,69,70].

Although photothermal speckle imaging demonstrated lower sensitivity in this study, it remains a valuable complementary approach, particularly in scenarios where colorimetric model training is limited or where image quality may be compromised. Future research should focus on optimizing the system and algorithms, such as utilizing higher-powered lasers and enhancing speckle contrast techniques, to further improve the LOD. Incorporating machine learning algorithms could be applied to refine photothermal analysis, enhancing sensitivity and detection accuracy.

Integrating both colorimetric and photothermal modalities into a unified platform could combine the strengths of each method, potentially improving reliability across diverse testing environments. This dual-monitoring system has the potential to be developed into a portable, user-friendly diagnostic device. The benchtop system presented in this study serves as a proof-of-concept platform, with potential for translation into a portable biosensor using low-cost microcontrollers and compact imaging modules. Integration with a smartphone and a user-friendly interface would further support its application in resource-limited setting, enabling accessible pathogen screening.

All experiments in this study were conducted in a controlled laboratory environment to ensure consistency and reproducibility. However, for the system to be effectively deployed in real-world settings, validation under realistic field conditions is essential. While this study employed data augmentation techniques to simulate variable lighting, these do not fully capture the complexity of field environments. Therefore, future studies should aim to evaluate the system’s robustness under field-relevant conditions to further assess its practical applicability and reliability.

To improve model generalizability, future work should consider incorporating additional augmentation techniques that simulate real-world imaging conditions such as motion blur, shadows, and image noise, which were not simulated in our current dataset. These techniques have been explored in other image analysis studies for LFA image analysis [71]. Incorporating such augmentations would help build a more comprehensive training dataset that better reflects field conditions.

Additionally, future studies should include more comprehensive testing involving non-target organisms and a wider range of food matrices. In this study, PBS was used as the negative control. Assessing whether non-target organisms produce a detectable signal, even in the absence of visible test line, can evaluate the risk of false positives. Different food matrices would help assess how differences in texture, color, and composition affect test line visibility and signal quality. Finally, developing quantitative calibration curves remains a key area for future research.

Rather than competing with gold-standard detection methods, this work aims to upgrade existing technologies by leveraging the strengths of LFAs while addressing their limitations through signal enhancement. With continued development, this system could contribute to the early detection and prevention of foodborne illnesses, ensuring safer food consumption and reducing public health risks.

## Figures and Tables

**Table 1:** Summary of regression model performance metrics. Pseudo-R^2^ values, Aikaike Information Criterion (AIC), and Bayesian Information Criterion (BIC) are presented.

Model	Pseudo-R^2^	AIC	BIC
Linear	0.1280	7.05 × 10^5^	7.05 × 10^5^
Polynomial (deg = 2)	0.1455	6.96 × 10^5^	6.96 × 10^5^
Logistic	0.2190	6.16 × 10^5^	6.16 × 10^5^

**Table 2:** Summary of regression model performance metrics with LASSO regularization. Pseudo-R^2^ values, AIC, and BIC are presented.

Model	Pseudo-R^2^	AIC	BIC
Linear with LASSO	0.126	8.51 × 10^5^	8.51 × 10^5^
Polynomial (deg = 2) With LASSO	0.1365	8.34 × 10^5^	8.34 × 10^5^
Logistic with LASSO	0.2175	7.62 × 10^5^	7.62 × 10^5^

## Data Availability

All data and code supporting the findings of this study are publicly available in the following GitHub repository: https://github.com/jully-blackshare/biosensor-colorimetric-photothermal (accessed on 7 August 2025).
